# A Lightweight and Accurate UAV Detection Method Based on YOLOv4

**DOI:** 10.3390/s22186874

**Published:** 2022-09-11

**Authors:** Hao Cai, Yuanquan Xie, Jianlong Xu, Zhi Xiong

**Affiliations:** Department of Computer Science, Shantou University, Shantou 515041, China

**Keywords:** object detection, UAV detection, deep learning, depth-wise separable convolution

## Abstract

At present, the UAV (Unmanned Aerial Vehicle) has been widely used both in civilian and military fields. Most of the current object detection algorithms used to detect UAVs require more parameters, and it is difficult to achieve real-time performance. In order to solve this problem while ensuring a high accuracy rate, we further lighten the model and reduce the number of parameters of the model. This paper proposes an accurate and lightweight UAV detection model based on YOLOv4. To verify the effectiveness of this model, we made a UAV dataset, which contains four types of UAVs and 20,365 images. Through comparative experiments and optimization of existing deep learning and object detection algorithms, we found a lightweight model to achieve an efficient and accurate rapid detection of UAVs. First, from the comparison of the one-stage method and the two-stage method, it is concluded that the one-stage method has better real-time performance and considerable accuracy in detecting UAVs. Then, we further compared the one-stage methods. In particular, for YOLOv4, we replaced MobileNet with its backbone network, modified the feature extraction network, and replaced standard convolution with depth-wise separable convolution, which greatly reduced the parameters and realized 82 FPS and 93.52% mAP while ensuring high accuracy and taking into account the real-time performance.

## 1. Introduction

With the rapid development of the UAV field, the number of UAVs used for military, commercial or recreational purposes is increasing day by day. This situation poses a serious threat to people’s privacy and security when cameras or weapons are installed on UAVs. For example, a drone collided with a Lufthansa jet near Los Angeles International Airport (LAX) on 29 March 2016, sparking concerns about the safety of government buildings, air traffic and other facilities worries [1]. UAVs near an airport could compromise the safety of the aircraft [2], or UAVs may carry bombs or dangerous chemicals in terrorist attacks.

Nevertheless, real-time surveillance is a cumbersome process, but it is absolutely essential to detect promptly the occurrence of adverse events or conditions. To that end, many challenging tasks arise such as object detection, classification, multi-object tracking and multi-sensor information fusion [3].

At present, the technologies used for the rapid detection and classification of UAVs mainly include radar, sound detection and visual detection. However, these methods are too complicated and require expensive computing costs such as the use of radar and infrared to detect UAVs [4]. Although radar and infrared equipment have high range accuracy, there are many blind spots, and if the frequency band is exceeded, it is difficult to be detected; i.e., the characteristics of humans and machines are analyzed, and it is impossible to distinguish between UAVs and birds. Another disadvantage of radar detection is its high cost and poor flexibility, which are inconsistent with the growing detection needs of UAVs [5].

Sound detection is sensitive to ambient noise, especially in noisy areas, and wind conditions can affect detection performance [6]. In addition, sound detection also requires the acoustic feature database of different UAVs for training and testing. In order to achieve low-cost UAV detection, although some people use deep learning image detection to detect UAVs, the amount of model parameters used is large, and these methods are difficult to achieve real-time detection of UAVs. Given these real-world resource constraints, model efficiency becomes increasingly important for object detection. This paper aims to find a lightweight model to achieve the real-time and accurate detection of UAVs. The low cost of visual inspection depends on the cameras and optical sensors used.

The localization and classification of UAVs by cameras belongs to the category of deep learning object detection. The field of object detection has been developed for more than 20 years. From the early traditional methods to today’s deep learning methods, the accuracy is becoming higher and higher, and the speed is becoming faster and faster thanks to the continuous development of deep learning and other related technologies. The task of object detection is to find objects of interest in an image or video and simultaneously detect their location and size. Different from image classification tasks, object detection not only solves the classification problem but also solves the localization problem [7]. With the rapid development of deep learning [8], more powerful tools have emerged that can learn to extract deeper feature information from images. The model of object detection mainly includes two parts: the detection head and the backbone network. The function of the backbone network is to extract the features of the input image, and the function of the detection head is to output the location and classification of the target in the image. The backbone feature extraction network mainly includes VGG-16 [9], Resnet-50 [10], CSPDarknet-53 [11], etc.

In this paper, in order to improve the detection and recognition speed of UAVs, it is necessary to reduce the number of parameters of the model. We use MobileNet as the backbone network, modify the feature extraction network and prediction network, and replace all standard convolutions in the model with depth-wise separable convolutions. Experiments show that introducing the feature extraction network MobileNet as the backbone network achieves better efficiency than previously commonly used networks such as VGG-16 or Resnet-50. It achieves better accuracy with fewer parameters. MobileNet [12,13,14,15] is a lightweight neural network proposed by Google, which is characterized by few parameters, high speed and low memory usage. It can be used as the backbone feature extraction network of the object detection network to perform preliminary feature extraction on the input features. It has excellent feature extraction effects while reducing network parameters.

The main contribution of this work is summarized as follows.

- On the basis of YOLOv4 [16], we use MobileNet as its backbone network, modify the feature extraction network and prediction network, and replace all standard convolutions with depth-wise separable convolutions, which greatly reduces parameters and achieves high real-time performance.
- We apply some widely recognized object detection methods, such as SSD [17], YOLOv4, and Faster R-CNN [18], to UAV detection. In order to improve the detection speed while maintaining the accuracy, we finally propose a lightweight and accurate UAV detection method based on YOLOv4.

## 2. Related Work

In this section, we mainly review the methods used to detect UAVs and the challenges encountered before, outline the deep learning object detection algorithm, and discover the scarcity of current public UAV datasets.

### 2.1. Methods of Detecting UAVs

Recently, many technical articles provide methods for UAV detection; however, most of these reviews have both advantages and disadvantages as summarized in Table 1.

### 2.2. Object Detection Algorithm

Currently, while object detection has been successfully applied to general category datasets, it remains a tough challenge for UAV detection tasks. Particularly in cloudy weather, light severely affects the quality of aerial UAV images, resulting in poor visibility, low contrast and color distortion. At the same time, the complex aerial environment and distractions such as birds make UAV detection even more difficult.

As the different object detection models may require different input sizes, the UAV detection task usually includes image pre-processing and object detection. Image pre-processing, i.e., changing the input size and image enhancement, aims to increase the amount of training data and improve the generalization ability of the model. The object detection framework includes four main parts including input, backbone, neck and head. The backbone is a network that extracts features from the objects of interest in the input image, such as VGG-16 [9], Resnet-50 [10], as well as MobileNet (V1, V2, V3) [12,13,14,15], DenseNet [20], CSPDarknet-53 [11], etc. The necks are usually located between the backbone network and the output layer, such as spatial pyramid pooling (SPP) [21], feature pyramid network (FPN) [22], path aggregation networks (PANets) [23], etc., which enhance feature maps that contain both rich semantic information and deterministic location information. The detection head is to find out the classification and localization feature of the object, which can be generally classified into anchor-based and anchor-free mechanisms in the two major representative algorithms, one-stage and two-stage algorithms, as shown in Table 2.

At present, although object detection algorithms are widely used in many fields, there is relatively little research applied to UAV detection; moreover, most of these algorithms require a large number of parameters, making it difficult to combine real-time and high accuracy in drone detection. So, our work focuses on the application of improved object detection algorithms to UAV detection. In this paper, we focus more on how to optimize the model and reduce the number of parameters of the model to achieve accurate detection of UAVs in real time while maintaining a high accuracy rate.

### 2.3. UAV Dataset

Datasets are the most important resource in every field of research and can contribute to the development of a field. There are already some UAV datasets that are suitable for UAV detection. However, most of them are either private or have only a small amount of data.

**The Anti-UAV Dataset** [27] is a non-public experimental dataset, including three experimental models and 49 experimental videos. All videos are annotated by the KCF tracking model [28].

**The USC-GRAD-STDdb** [29] provides a series of annotated videos for small object detection. Small objects include UAVs, boats, vehicles, people, and birds. The image size is 1270 × 720 pixels. The USC-GRAD-STDdb is one of the few public datasets with UAV imagery. However, the datasets are mostly small objects that are difficult for people to recognize, and it is not suitable for use as a dataset for this study.

Generally speaking, there are very few publicly available UAV datasets. However, almost object detection methods are data driven and depend on a large-scale, well-labeled dataset [30]. To solve this problem, it is necessary to make a public dataset related to UAV.

## 3. Methodlogy

This part of the paper mainly describes the UAV detection framework as well as the data acquisition and processing of UAV detection. Finally, a lightweight and accurate UAV detection method based on YOLOv4 is proposed.

### 3.1. Detection Framework

**Figure 1:**
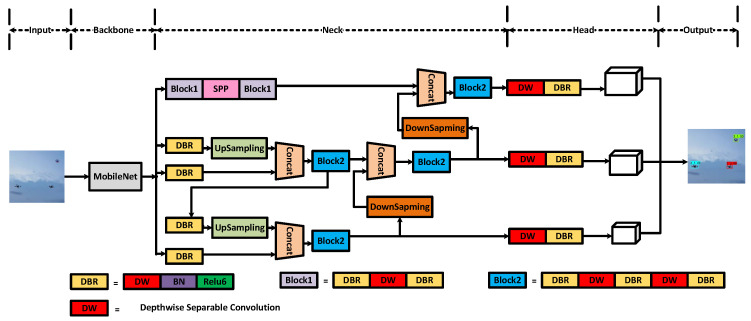
The structure of lightweight and accurate UAV detection method based on YOLOv4.

Figure 1 demonstrates the improved structure of the lightweight and accurate UAV detection method based on YOLOv4. The main improvements of our work are in the parts of backbone, neck and YoloHead.

In order to achieve real-time and accurate detection of UAVs and solve the problem of too many parameters in the traditional UAV detection model, this paper optimizes YOLOv4 and replaces the original CSPDarknet-53 with the MobileNet series of networks for the model’s backbone feature extraction network. To further decrease the number of model parameters, the neck and YoloHead structures were rebuilt with depth-wise separable convolution; i.e., we replaced the normal convolution in all the convolution blocks in SPP, PANet and YoloHead with a depth-wise separable convolution.

In all networks, 3 × 3 depth-wise separable convolution and 1 × 1 standard convolution are used to replace the 3 × 3 standard convolution in the network, which greatly reduces the model parameters and maintains a high-precision UAV detection effect. In the process of model training, firstly, data enhancement is performed on the input image, such as mosiac processing. Then, we use convolution to downsample the input image, extract image features, and obtain a preliminary feature layer. Part of the feature layers are upsampled to obtain effective feature maps with high resolution. Next, the effective feature layer and part of the preliminary feature layer perform feature fusion, the result is sent to the prediction head, and the prediction frame of the picture is adjusted and compared with the real frame. During the prediction process, the prediction frame and score of each type of UAV are taken out, and the position and score of the frame are used for non-maximum suppression, and finally, the prediction result is obtained.

### 3.2. Data Acquisition and Processing for UAV Detection

**Figure 2:**
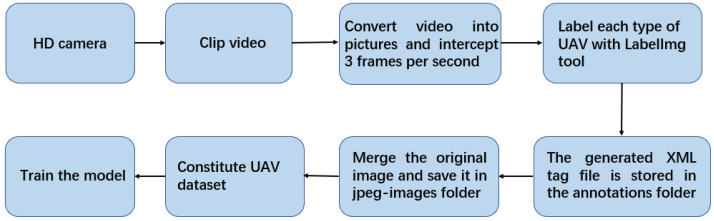
Flowchart for making a UAV dataset.

The production process of the UAV dataset is shown in Figure 2. Each image is carefully annotated; this is a high-quality dataset. There are a total of four types of UAVs in the experiment: namely, No. 1, No. 3, No. 4 and No. 5. The images in the UAV dataset are extracted from videos at a frequency of about ten frames per second, and the pictures are resized to 1280 × 720 resolution. In order to distinguish the recognition of the UAV in different scenes, the sky contains sunny and cloudy backgrounds when shooting. In addition, 6 videos are included in the dataset as validation and test data.

After labeling the original image in the UAV dataset and generating the label file, it is further processed by mosiac data enhancement and then input to the model for training. Mosiac data enhancement, i.e, image stitching, distortion, pixel adjustment, background brightness adjustment, etc., can enrich the background of the detection target. The data of four images can be calculated at one time during model calculation. Image augmentation creates new training samples from existing training data.

### 3.3. Depth-Wise Separable Convolution

**Figure 3:**
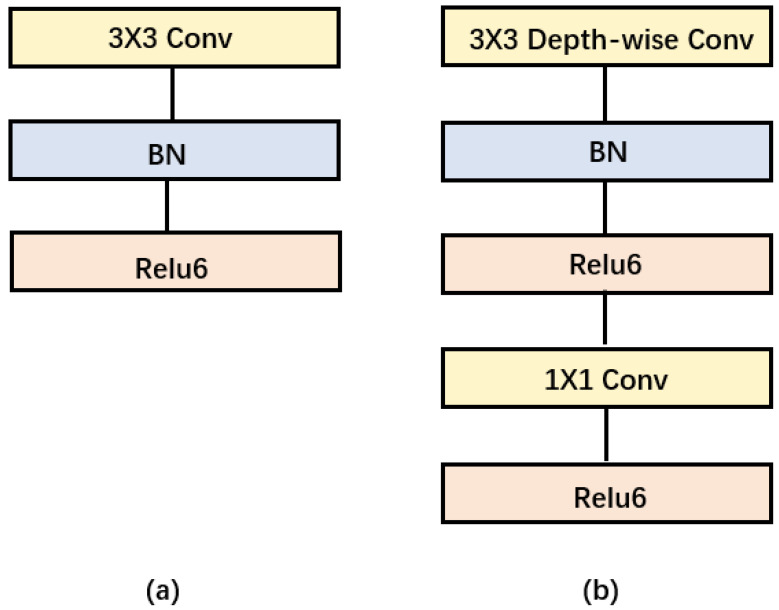
(**a**) Convolution blocks with standard convolution. (**b**) Convolution blocks with depth-separable convolution.

Depth-wise separable convolution is the basic component of MobileNet, but in the real application, batchnorm [31] and the ReLU activation function [32] will be used, so the basic structure of the depth-wise separable convolution is shown in Figure 3. Depth-wise separable convolutions are actually factorized convolutions, which can be decomposed into two smaller operations: depth-wise convolution and point-wise convolution. Depth-wise convolution is different from standard convolution. For standard convolution, its convolution kernel is used on all input channels, while depth-wise convolution uses different convolution kernels for each input channel; that is, one convolution kernel corresponds to one input channel, so depth-wise convolution is a depth-level operation. The point-wise convolution is actually a standard convolution and uses a 1 × 1 convolution kernel. For depth-wise separable convolution, it first uses depth-wise convolution to convolve the different input channels separately and then uses point-wise convolution to combine the above outputs. In fact, the overall effect is similar to that of standard convolution, but it will significantly reduce the computational and model parameters.

**Figure 4:**
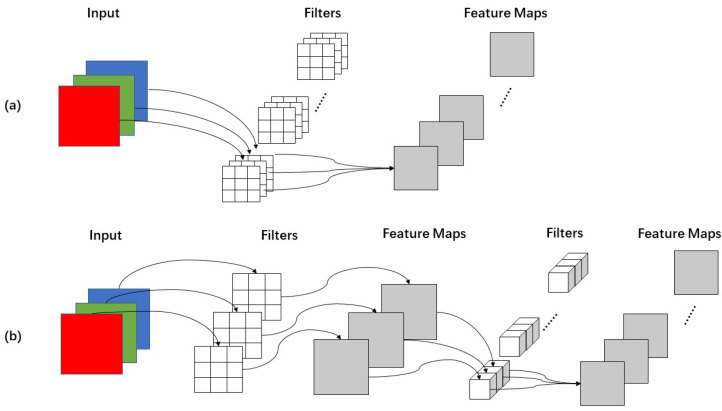
The specific process of convolution. (**a**) standard convolution, (**b**) depth-wise separable convolution.

Figure 4 gives the specific convolution process for standard convolution and depth-wise separable convolution. The input layer is supposed to be a three-channel color image of size 64 × 64 pixels. After a convolution layer containing N filters, the final output is 4 feature maps with the same size as the input layer. For standard convolution, the convolution layer has a total of N filters, each of which contains three kernels, each of size 3 × 3. So, the number of parameters in the convolution layer is N × 3 × 3 × 3 = 27 N. For depth-wise separable convolution, a three-channel image is computed to generate three feature maps, and a filter contains only one kernel of size 3 × 3, and the number of parameters in the convolution part is 3 × 3 × 3 = 27. In point-wise convolution, the size of the convolution kernel is 1 × 1 × M, with M being the depth of the previous layer, and the number of multiplications is 1 × 1 × 3 × N. Therefore, the parameters of the depth-wise separable convolution is 27 + 3 N. If N = 4 in this case, then depth-wise separable convolution has about 1/3 the number of parameters of conventional convolution, which indicates that the depth-wise separable convolution significantly reduced the computation cost and number of parameters.

### 3.4. Lightweight and Accurate UAV Detection Method Based on YOLOv4

**Figure 5:**
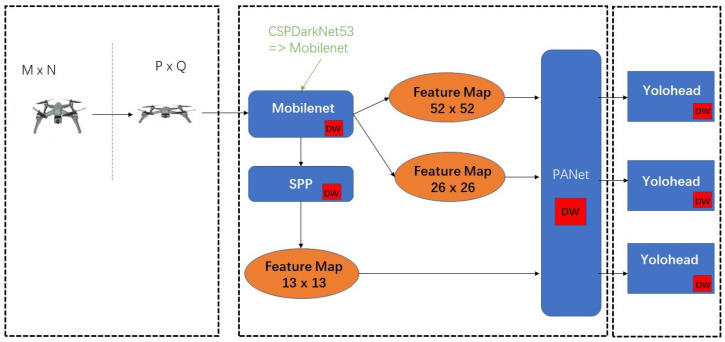
Lightweight YOLOv4 structure diagram.

The network model diagram of the lightweight YOLOv4 is shown in Figure 5. The input size of the image will be resized to 416 × 416. The backbone network of the model adopts the MobileNet series network to replace CSPDarkNet-53. The backbone network performs preliminary feature extraction, and three preliminary effective feature layers can be obtained. Then, by enhancing the feature extraction network PANet (replacing the standard convolution with depth-wise separable convolution), the three preliminary effective feature layers are feature-fused to obtain three more effective effective feature layers. The prediction frame (replacing the standard convolution with depth-wise separable convolution) is obtained by adjusting the prior frame corresponding to each effective feature layer by convolution. It is also necessary to perform score sorting and non-maximum suppression screening for each prediction box and finally obtain the prediction result.

**Figure 6:**
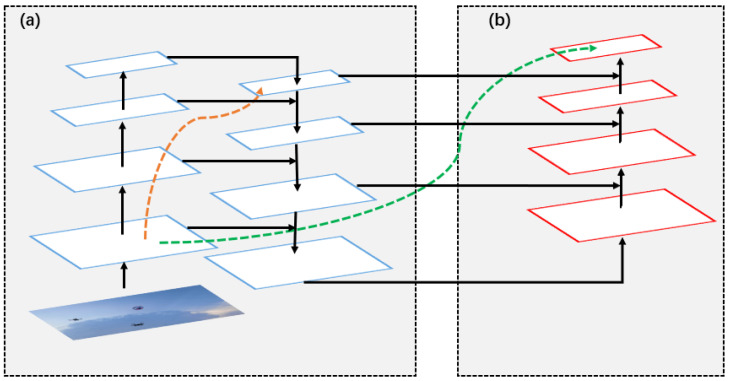
The structure of the original PANet.

1. SPP [21] and PANet [23] are enhanced feature extraction networks. PANet is an instance segmentation algorithm in 2018, and its specific structure is shown in Figure 6. In YOLOV4, it mainly uses the PANet structure on three effective feature layers, which repeatedly extracts features. The PANet enhanced feature extraction network is quite large, and most of the parameters of the YOLOv4 model are generated by this network. As can be seen in Figure 6, it has a very important role to play in the repetitive extraction of features. In (a) is the traditional feature pyramid structure. After completing the feature extraction from the bottom to the top of the feature pyramid, the top-to-bottom feature extraction in (b) needs to be implemented. By modifying PANet, this paper uses 3 × 3 depth-wise separable convolution and 1 × 1 standard convolution to replace the 3 × 3 standard convolution in the network, which greatly reduces the number of parameters of the model.
2. The activation function uses Mish [33]. The formula for the Mish activation function is shown in Equation (1).
  (1) $$Mish=x*tanh(ln\left(1+e^{x}\right))$$
3. The prediction network YoloHead uses the obtained features to make predictions. The CIoU (Complete-IoU) algorithm [34] is used in the prediction network. IoU (Intersection over Union) is a concept of ratio, which is insensitive to the scale of the target object. However, the regression loss optimization and IoU optimization of the commonly used BBox are not completely equivalent, and the ordinary IoU cannot directly optimize the non-overlapping part. Therefore, using CIoU, CIoU takes the center point distance between the target box and the anchor box, the overlapping area and the aspect ratio into account in the calculation. The calculation equation is shown in (2) and (3).
  (2) $$IoU=\frac{A\cap B}{A\cup B}$$
  (3) $$CIoU=IoU-\frac{\rho^{2}\left(b,b^{gt}\right)}{c^{2}}-\alpha \upsilon$$
  where *A* is the area of the prediction box, and *B* is the area of the ground truth box. $b,b^{gt}$ represents the center points of the predicted box and the ground truth box, respectively, $\rho^{2}\left(b,b^{gt}\right)$ is the Euclidean distance between the center points, and c is the diagonal length of the smallest enclosing box covering the two boxes. α is a positive trade-off parameter, and υ measures the consistency of the aspect ratio.
  The consistency of the aspect ratio can be defined as
  (4) $$\upsilon =\frac{4}{\pi^{2}}{\left(\arctan\frac{w^{gt}}{h^{gt}}-\arctan\frac{w}{h}\right)}^{2}$$
  where $w^{gt}$ is the width of the ground truth box and $h^{gt}$ is the height of the ground truth box; *w* is the width of the prediction box and *h* is the height of the prediction box.
  The trade-off parameter α is defined as
  (5) $$\alpha =\frac{\upsilon }{1-IoU+\upsilon }$$
  Then, the CIoU loss function can be defined as
  (6) $$Loss_{CIoU}=1-IoU+\frac{\rho^{2}\left(b,b^{gt}\right)}{c^{2}}+\alpha \upsilon$$

## 4. Experiments

Since the main contribution of this work is to develop a lightweight UAV accurate and real-time detection model, we aim to answer the following research questions experimentally:

**RQ1** How effective is the lightweight YOLOv4? After replacing standard convolution with depth-wise separable convolution, is the amount of model parameters greatly reduced?

**RQ2** How does the lightweight YOLOv4 perform compared to other image detection algorithms?

**RQ3** How do hyperparameter batch-size and learning rate lr affect performance, and how should we choose optimal values?

Next, we first describe the dataset for the experiments and the processing of the dataset. We then report the results by sequentially answering the above research questions through comparative experiments.

### 4.1. UAV Dataset and Data Pre-Processing

**Figure 7:**
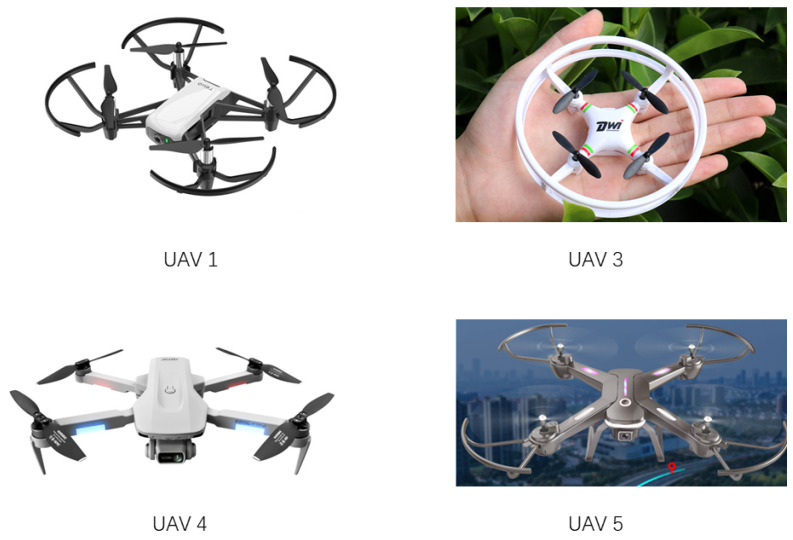
Four different models of UAVs.

**Figure 8:**
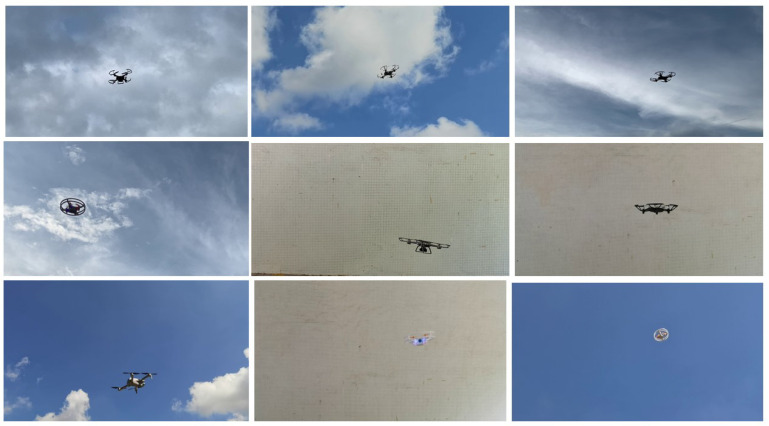
Original images of the UAV dataset.

In view of the problem that there are few publicly available UAV datasets, this study produced and released different types of UAV datasets in different contexts. In the experiments, based on the pytorch framework, various models were trained to locate and identify four kinds of small UAVs shown as Figure 7. Detailed descriptions of the four kinds of UAVs in the dataset are shown in Table 3. In the experiment, they were named as No. 1, No. 3, No. 4, and No. 5 UAVs, and some pictures from the dataset are shown in Figure 8. 20,356 UAV images are trained, features are extracted, and regression prediction of UAV is realized. Detection and results of multiple UAVs were achieved using Faster-RCNN, EfficientDet, SSD and YOLOv4 and their models using different CNN frameworks. In order to verify that the object detection model can achieve an efficient detection of UAVs, we divided the self-made UAV data set according to the ratio of 9:1, 90% as the training set and 10% as the test set. The training set is the data sample used for model fitting and used to debug the parameters in the network. The test set is used to check the training effect. Finally, we compare the results obtained by the optimized lightweight YOLOv4 model with other models using the test set.

**Figure 9:**
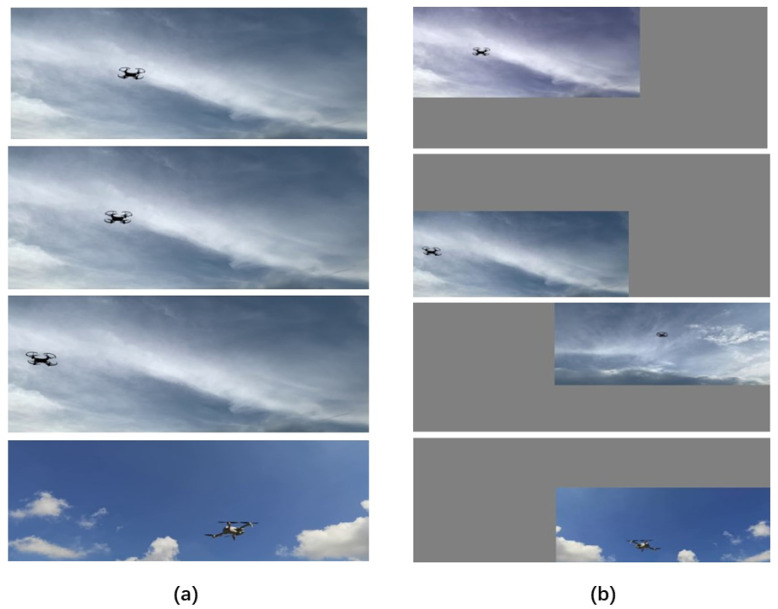
The process of mosiac data enhancement. (**a**) The four original images of the UAV dataset, (**b**) Images that have been flipped, scaled, colour gamut transformed, and placed in four orientations.

**Figure 10:**
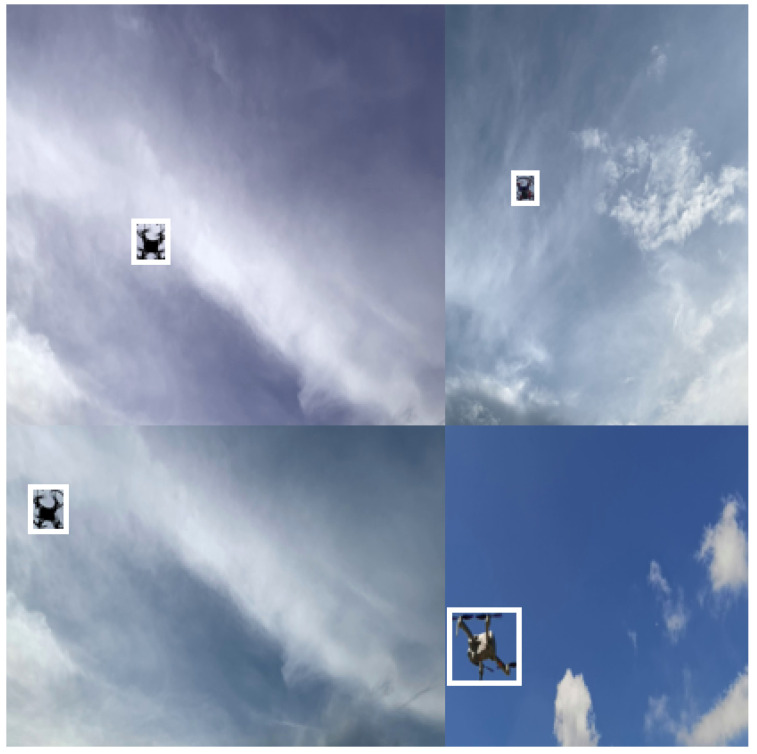
The result of merging images and combining boxes.

We aimed to increase the amount of training data, improve the generalization ability of the model, and improve the robustness of the model. In Figure 9, four randomly selected images from the dataset are processed by mosiac data enhancements to form a single image that is richer in feature information. The results of processing the original images of the dataset is shown in Figure 10.

### 4.2. Evaluation Metrics for Model Performance

For the deep learning network model, it is hoped that it has high accuracy, fast speed and small memory. Therefore, quantitative indicators are needed to evaluate these performances. Common indicators are: mAP (accuracy indicator), FPS (speed indicator), and model parameter size (memory size indicator). Among them, mAP (mean Avearage Precision) refers to the average value of AP for each category, and AP refers to the area of the PR curve (precision and recall relationship curve). From the perspective of prediction results, precision describes how many positive examples predicted by the classifier are real positive examples, that is, how many positive examples predicted by the classifier are accurate. From the perspective of real results, recall describes how many real positive examples in the test set are selected by the classifier, that is, how many real positive examples are recalled by the classifier.

(7) $$Precision=\frac{TP}{TP+FP}$$

(8) $$Recall=\frac{TP}{TP+FN}$$

where *TP*, *FP*, and *FN* indicate true positive, false positive, and false negative, respectively.

**Figure 11:**
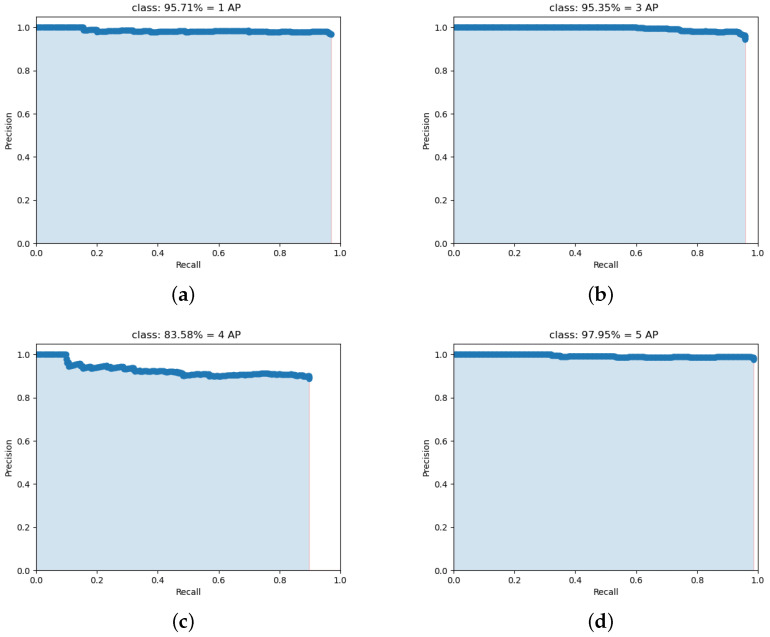
The PR curves of four types of UAVs. (**a**) The PR curves of UAV 1; (**b**) The PR curves of UAV 3; (**c**) The PR curves of UAV 4; (**d**) The PR curves of UAV 5.

Precision and recall reflect two aspects of classifier performance, and a single index cannot comprehensively evaluate the performance of a classifier. In general, the higher the precision, the lower the recall; conversely, the higher the recall, the lower the precision. In order to balance the influence of precision and recall, and to evaluate a classifier more comprehensively, the comprehensive index of AP is introduced. Taking precision as the *y*-axis and recall as the *x*-axis will form a precision/recall curve. We compute the AP as the area under this curve by numerical integration. In the lightweight and accurate UAV detection method based on YOLOv4, the PR curves of four types of UAVs are shown in Figure 11.

(9) $$AP=\frac{\left(r_{2}-r_{1}\right)p_{2}+\left(r_{3}-r_{2}\right)p_{3}+\cdots +\left(r_{i+1}-r_{i}\right)p_{i+1}}{n}=\frac{1}{n}\sum_{i=1}^{n-1}\left(r_{i+1}-r_{i}\right)p_{i+1}$$

where $r_{1},r_{2},\ldots ,r_{n}$ is the corresponding recall value in the X coordinate axis, and $p_{1}$, $p_{2}$, *…*, $p_{n}$ is the corresponding precision value in the Y coordinate axis.

Therefore, the target detection mAP calculation method is as follows: Given a set of IOU thresholds, under each IOU threshold, calculate the AP of K categories, and average them as the detection performance under this IOU threshold. So, the final performance evaluation mAP is shown in Equation (10).

(10) $$mAP=\frac{1}{K}\sum_{i=1}^{K}AP_{i}$$

### 4.3. Improvements in Model Size (RQ 1)

A large part of the parameters in YOLOv4 come from the PANet network, which uses features from all layers and lets the network decide which are useful. YOLOv4 mainly uses the PANet structure on three effective feature layers. The important feature of PANet is the repeated extraction of features. After using the traditional feature pyramid structure to complete the feature extraction from the bottom to the top of the feature pyramid, it is also necessary to implement the feature extraction from the top to the bottom. In this paper, by replacing CSPDarkNet-53 with the MobileieNet series of networks, and modifying the ordinary convolution of extracted features in PANet into a depth-wise separable convolution, the parameter amount is greatly reduced. As shown in the Table 4, in the original YOLOv4, CSPDarkNet-53 is used as the backbone network, and PANet is in the form of standard convolution. The overall parameter of the model is 64.1M, while in the optimized lightweight YOLOv4, its backbone network is MobileNetv1, and the standard convolution for extracting features in the PANet network is replaced by a depth-wise separable convolution, and the overall parameter volume of the model is reduced to 10.9M. The one-stage methods, such as SSD, EfficientDet, YOLOv4, etc., are applied to the detection of UAVs, and the accuracy of various models and the operation speed of the models are obtained through comparative experiments. Although the number of parameters of the SSD model is small, its detection accuracy is low, while the traditional YOLOv4 has high detection accuracy, but the model parameters are too large to meet the real-time requirements.

In the lightweight YOLOv4, we replace the backbone feature extraction network and use the MobileNet series of networks. In particular, by replacing the standard convolution method in the enhanced feature extraction network PANet in YOLOv4 with a depth-wise separable convolution, the model parameters are reduced by 5.88 times, and the accuracy is as high as 93.14%.

### 4.4. Performance Comparison (RQ 2)

The object detection models in the comparative experiments include Faster-RCNN, SSD, EfficientDet, YOLOv4 and the optimized YOLOv4. Through the comparative experiments of five different detection models, a model with real-time and accurate detection of UAVs is found. In order to achieve the best training results, different input sizes are often required, so the input size of the image needs to be changed before the image is input into the feature extraction network. According to the parameters of the model, the number of frames per second processed UAV pictures and the average accuracy of the three indicators, by adjusting the parameters and optimizing the model, we can repeat the experiment and obtain the following experimental results, as shown in Table 5.

In terms of speed, based on the UAV data set, the vgg-16 SSD detection method achieved the best performance in terms of speed: fps reached 88, but the accuracy rate was only 79.25%, which could not satisfy the accuracy of UAVs detection. In addition, the lightweight YOLOv4 model with the MobileNet series as the backbone network has about six times less parameters than the original YOLOv4, and the detection speed is two times faster. The fps of the lightweight YOLOv4 with MobileNetv1 as the backbone feature extraction network reaches 82: that is, 82 frames of pictures can be processed in one second, which has very high real-time performance.

**Figure 12:**
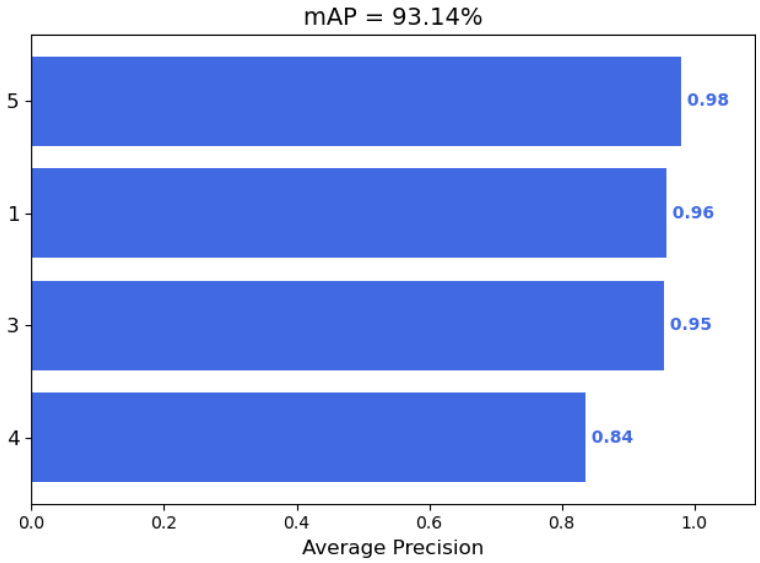
In the lightweight YOLOv4, with MobileNetv1 as the backbone network, the AP values of each category of UAVs are presented in the bar graph, and the final mAP is calculated.

In terms of accuracy, YOLOv4 with CSPDarkNet as the backbone feature extraction network has the best performance with an average accuracy of 96.02%. In contrast, the lightweight YOLOv4 still maintains a high accuracy rate despite a slight decrease. As shown in Figure 12, the lightweight YOLOv4 with MobileNetv1 as the backbone network achieves an mAP of 93.14%. In addition, the accuracy rates of MobileNetv2 and MobileNetv3 as the backbone feature extraction network reached 92.98% and 93.52%, respectively. This also shows that the optimized lightweight YOLOv4 has a very significant effect. Compared with several other target detection models, the lightweight YOLOv4 ensures high accuracy while taking into account real-time performance.

### 4.5. Hyperparameter Research (RQ 3)

**Figure 13:**
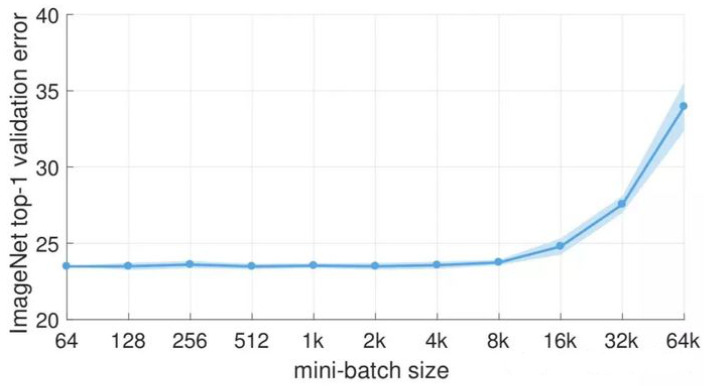
Batch size versus model performance.

There are two important parameters in the lightweight YOLOv4: batch size (batch-size) and learning rate (lr). Batch size (batch size) is an important parameter in machine learning, which indicates how many batches of images are read at one time. The learning rate determines the step size of the weight iteration, the learning rate directly affects the convergence state of the model, and the batch size affects the generalization performance of the model. In the process of model training, when we already have some pre-trained weights, and the part of the network to which these pre-trained weights are applied is common, such as the backbone network, then we can first freeze the training of these weights. Putting more resources into training the network parameters in the latter part can greatly improve both time and resource utilization. Wait until the following network parameters are trained for a period of time before unfreezing these frozen parts, and then train them all together. Therefore, training can be divided into two phases: freeze phase and unfreeze phase. In the freezing phase, the backbone feature extraction network of the model is frozen and will not change. At this time, the model training has fewer parameters and occupies less memory. Within a certain range, increasing the batch size can reduce the training time and help stabilize the convergence of the model training curve. However, as the batch size increases, the performance of the model decreases, and research [35] shows that the batch size that causes the performance drop is around 8000. In [36], it shows that the performance of the large batch size decreases because the training time is not long enough. The parameter updates under the same epochs are reduced, so a longer number of iterations is required. Considering the limitation of computing resources and the relationship between model performance and bach size as shown in Figure 13, we set the batch size to 128 in the model freezing training phase and set it to 64 in the unfreezing training phase.

**Figure 14:**
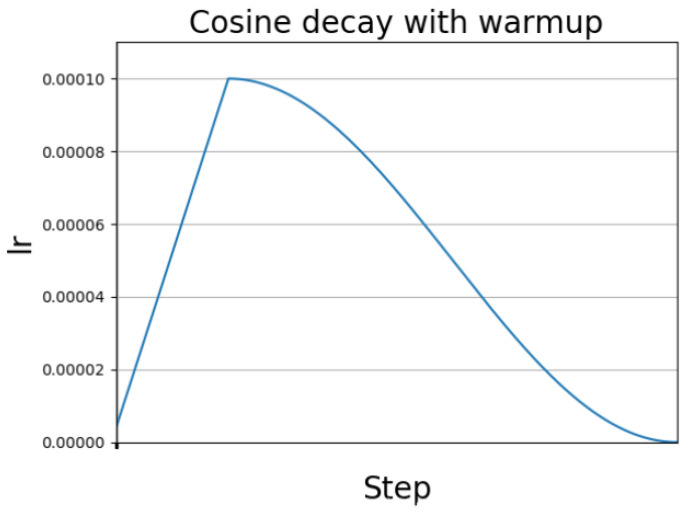
Learning rate cosine annealing decay.

The learning rate cosine annealing strategy is used in YOLOv4. In [37], it mainly introduces the stochastic gradient descent algorithm with restart (SGDR), which introduces the learning rate descent method of cosine annealing. Because our target optimization function may be multimodal, and there are multiple local optimal solutions in addition to the global optimal solution, the gradient descent algorithm may fall into a local minimum during training. At this time, we can suddenly increase the learning rate to avoid “Jump out” of the local minimum and find a path to the global minimum. This method is called stochastic gradient descent with restart, and its effect is shown in Figure 14. The influence of the learning rate on the performance of the model is reflected in two aspects: the first is the size of the initial learning rate, and the second is the transformation scheme of the learning rate.

In the model freezing training phase, we train for 50 generations. At this time, there are few parameters for model training. We can increase the learning rate (lr) to help the model adjust to the local optimal solution. In the model freezing phase of the experiment, after repeated experiments, it turns out that initializing the learning rate to 0.001 can achieve good results. In the thawing phase, we train for 50 generations, and the backbone feature extraction network of the model is no longer frozen. At this time, the model training has many parameters, which occupies a large amount of video memory, and all the parameters of the network will be changed. At this time, there are many parameters in the model training, and the batch size needs to be set smaller. At this time, the learning rate (lr) should also be set smaller to maintain the stability of the model. In the experiments we initialize the learning rate in the model unfreezing phase to 0.0001. The learning rate transformation scheme of the training process adopts the learning rate cosine annealing strategy.

**Figure 15:**
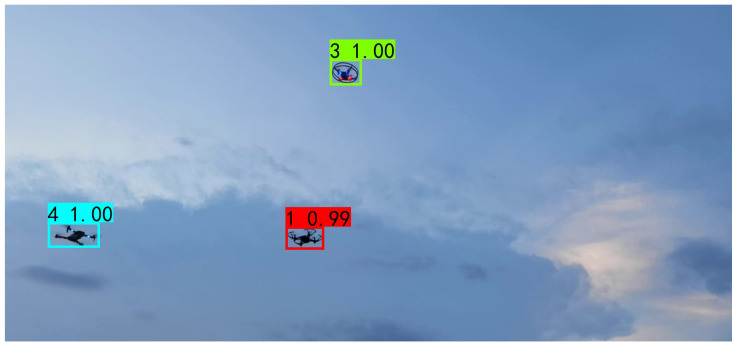
Visualization detection results of the lightweight and accurate UAV detection method based on YOLOv4.

The prediction renderings show that in the same scene, multiple UAVs can be accurately identified, and the model confidence reaches the highest value of 1.00. This is shown in Figure 15, when the background is a non-pure sky. Although the content of the picture is more complex, the UAV can be recognized accurately. The confidence level of the No. 1 machine is 0.99, the confidence level of the No. 3 machine is 1.00, and the confidence level of the No. 4 machine is 1.00.

## 5. Conclusions and Future Work

In this paper, we propose a lightweight and accurate UAV detection method based on YOLOv4. A dataset of UAV images was produced for this study. It contains four different types of UAVs, a total of 20,365 images with UAVs, and each image has been manually annotated with very high quality. Then, different deep learning object detection algorithms are used on this UAV dataset to identify UAVs. We utilized widely used object detection methods, such as SSD, Faster-RCNN, EfficientDet, and YOLOv4 as the baseline models for detecting UAVs. Through the comparative experiments of different models, the effects of different models on UAV recognition are obtained. The lightweight and accurate UAV detection method based on YOLOv4 achieved very good performance, the detection speed reached 82 fps, and the mAP reached 93.52%.

In the future, we will continue to improve the supplementary dataset by adding types of UAVs, for example by introducing large unmanned aircraft, enriching the context in which UAVs fly and introducing similar disturbing factors such as balloons and flying birds. We will further optimize detection by increasing the diversity and richness of the dataset. In addition, we will continue to tune hyperparameters and optimize the model to further improve the speed and accuracy of UAV detection.

## Figures and Tables

**Table 1:** Methods of detecting UAVs.

Methods of Detecting UAVs	Advantages	Disadvantages
Radar	Track UAVs over long distances, day/night, in all weather conditions and obtain various information (distance, speed, angle of arrival, micro-Doppler feature) [4].	Some weather echoes, ground car echoes and even insect echoes close to the antenna may cause great misleading. How to effectively distinguish birds from “low–slow–small” UAVs becomes an important challenge.
Acoustic	Works in low-visiblility environment. Low cost depending on the microphone used.	Difficulty identifying UAVs in noisy environments. Requres a dataset of sound signals of UAVs for training and testing [19].
Visual	Low cost depending on the employed cameras and optical sensors. Human evaluation of object detection results using the screen is simpler than otherwise.	Visibility is affected by dust, fog, clouds and time of day. A wide range of cameras and sensors need to be deployed.
Object detection algorithms	Track UAVs over long distances, day/night, in all weather conditions and obtain various information (distance, speed, angle of arrival, micro-Doppler feature).	Most models are relatively complex and require expensive computational costs, few algorithms combine high accuracy with real-time performance.

**Table 2:** Object detection methods of UAV detection.

Model	Backbone	Detection Head
Faster-RCNN [18]	Resnet-50	two stage
SSD [17]	VGG-16	one stage
EfficientDet [24]	Efficientnet [25]	one stage
YOLOv3 [26]	Darknet-53	one stage
YOLOv4 [16]	CSPDarknet-53	one stage

**Table 3:** Detailed description of the four kinds of UAVs in the dataset.

Annoation	Name of the UAV	Dimensions of the UAV (cm)	Number of Images
UAV 1	DJI Tello	9.8 × 9.25 × 5	5060
UAV 3	Dwi mini UAV	9.5 × 9.5 × 3	5017
UAV 4	4K photography UAV	22 × 18 × 5	5047
UAV 5	Dahan Frontier Y03 UAV	33 × 33 × 8	5232

**Table 4:** Comparison of experimental results with Mobienet series as backbone network.

Detection Head	Backbone	Input Size	Parms
YOLOv4	CSPDarknet	416 × 416	64.1 M
	MobileNetv1	416 × 416	41.0 M
	MobileNetv2	416 × 416	39.1 M
	MobileNetv3	416 × 416	40.0 M
Lightweight and accurate UAV detection method based on YOLOv4	MobileNetv1	416 × 416	12.7 M
	MobileNetv2	416 × 416	**10.9 M**
	MobileNetv3	416 × 416	11.8 M

**Table 5:** Comparison of experimental results of different models.

Detection Head	Backbone	Input Size	Parms	Speed (fps)	mAP
Faster-RCNN	Resnet-50	800 × 800	—	14	86.61%
SSD	VGG-16	300 × 300	24.2 M	88	79.25%
EfficientDet	efficinetd0	512 × 512	3.9 M	28	40.5%
YOLOv4	CSPDarknet-53	416 × 416	64.1 M	40	96.02%
Lightweight and accurate UAV detection method based on Yolov4	MobileNetv1	416 × 416	12.7 M	**82**	**93.14%**
	MobileNetv2	416 × 416	10.9 M	70	93.41%
	MobileNetv3	416 × 416	11.8 M	62	93.52%
